# The effects of solid-state fermentation on the content, composition and *in vitro* antioxidant activity of flavonoids from dandelion

**DOI:** 10.1371/journal.pone.0239076

**Published:** 2020-09-15

**Authors:** Na Liu, Min Song, Naifeng Wang, Yuan Wang, Ruifang Wang, Xiaoping An, Jingwei Qi

**Affiliations:** 1 College of Animal Science, Inner Mongolia Agricultural University, Hohhot, Inner Mongolia, China; 2 Inner Mongolia Herbivorous Livestock Feed Engineering and Technology Research Center, Hohhot, Inner Mongolia, China; Institute for Biological Research "S. Stanković", University of Belgrade, SERBIA

## Abstract

Dandelion (*Taraxacum officinale*), a common plant worldwide, is used as both a medicine and food. Fermentation is a food processing technology that has many advantages, such as low energy cost, changes in product characteristics, and enhanced product quality. The purpose of this study was to investigate the effect of solid-state fermentation (SSF) on the content, composition and antioxidant activity of dandelion flavonoids. Response surface methodology was used to optimize dandelion fermentation conditions. Under optimized conditions, the maximum flavone concentration was 66.05 ± 1.89 mg/g. The flavonoid content of the crude extract from fermented dandelion (FDF) was 183.72 ± 2.24 mg/g. The flavonoid compounds in the crude extracts were further identified by UPLC-ESI-MS/MS. A total of 229 flavonoid compounds were identified, and 57 differential flavonoids (including 27 upregulated and 30 downregulated flavonoids) between FDF and the crude extract of unfermented dandelion (DF) were observed, of which 25 were annotated to metabolic pathways. FDF displayed superior antioxidant activity to that of DF in *in vitro* DPPH radical-scavenging and reducing power assays. The favorable results of our investigation could provide a new way for the exploitation and utilization of dandelion, which could be promising for its application as an antioxidant and functional food additive with flavonoids as ingredients.

## 1. Introduction

Dandelion (*Taraxacum officinale*) is a perennial herb belonging to the Asteraceae family that contains various bioactive compounds, including flavonoids, phenolic compounds, alkaloids, and terpenes, and is widespread throughout the world [1,2]. This plant is traditionally consumed for food and medicinal purposes due to its hepatoprotective, choleretic, diuretic, anticarcinogenic, anti-inflammatory, and antioxidant properties [3,4]. Additionally, dandelion and its extracts are recognized as safe and have no reported negative effects on humans [5,6]. Dandelion has been considered a natural remedy for many centuries in the treatment of liver inflammatory diseases, gastrointestinal ailments, osteoarthritis, cancer, eye diseases, eczema and anemia, but few efforts have been made to improve compound yields from dandelion [7]. It is very important to find a way to increase the bioavailability of bioactive substances from dandelion. Solid-state fermentation (SSF) could offer a unique process development for value addition and utilization of dandelion [8].

Flavonoids are a large group of plant secondary metabolites and include flavanones, flavonol derivatives of quercitrin and myricitrin, anthocyanins, flavones, and chalcones [9]. Previous reports have revealed that flavonoids possess strong antioxidant activity due to their ability to scavenge radicals and participate in antioxidant reactions [10–12], and they can be used to protect the human body from chronic diseases [13] and inflammatory disease effects [14]. Moreover, flavonoids are also an integral part of diets for both humans and animals because they cannot synthesize flavonoids [9].

In recent years, numerous reports have documented the contents of flavonoids or phenolic compounds in dandelion and their characterization and biological activities, and there was an association between the chemical composition and bioactivity of dandelion [5, 15–18]. However, the effects of SSF on the content, composition and antioxidant activity of flavonoids from dandelion have not been studied. Fermentation is a food processing technique that has many advantages, such as low energy cost, changed product characteristics, and enhanced quality. During fermentation, a variety of enzymes, including lipase, glucoamylase, protease and amylase, produce and promote the release or synthesis of bioactive compounds from the substrate. Optimization of the fermentation conditions to maximize flavonoids in dandelion is important for future research, development and application of dandelion.

In the current study, we investigated the content, composition and antioxidant activity of dandelion flavonoids after being subjected to SSF processing. First, response surface methodology was applied to optimize the fermentation conditions (including fermentation time, fermentation temperature, inoculum concentration and moisture content) for dandelion flavonoid production. Then, under optimized conditions, the contents of flavonoids in the crude extracts of unfermented dandelion (DF) and fermented dandelion (FDF) were determined. The flavonoid compounds in the crude extracts before and after fermentation were further identified by UPLC-ESI-MS/MS. Through clustering analysis, principal component analysis (PCA), and partial least squares-discriminant analysis (OPLS-DA), different samples were clearly distinguished. Finally, the antioxidant properties of FDF and DF *in vitro* in DPPH radical and reducing power assays were evaluated. This study provides new insights into the flavonoids in fermented dandelion and a theoretical basis for the utilization of dandelion.

## 2. Materials and methods

### 2.1 Materials

Dandelion was grown and marketed from Hohhot city in the Inner Mongolia Autonomous Region of China. Dandelion was purchased from a local market (Hohhot, Inner Mongolia, China) and identified by Professor Zhaozhe Li of Inner Mongolia Agricultural University. The whole plant of dandelion was air-dried, milled through a 1.0 mm screen and used as a substrate for SSF. *Lactobacillus plantarum* (CGMCC No. 1.12934) and *Saccharomyces cerevisiae* (CGMCC No. 2.1190) were purchased from the China General Microbiological Culture Collection Center (Beijing, China).

2,2-Diphenyl-1-picryl-hydrazyl (DPPH) was purchased from Sigma Chemical Co. (St. Louis, USA). Butylated hydroxyanisole (BHA) was purchased from Sinopharm Chemical Reagent Co. (Beijing, China). Methanol, acetonitrile, ethanol and a standard mixture (Beijing, China) were of chromatographic grade. Other chemicals used in this study were of analytical grade.

### 2.2 Optimization of fermentation conditions and preparation of the FDF

SSF technology was adopted to ferment dandelion to produce flavonoids. Based on previous laboratory studies, *L*. *plantarum* and *S*. *cerevisiae* were mixed at a ratio of 3:7 and used for dandelion fermentation. For inoculation, *L*. *plantarum* (1.0×10^8^ CFU/mL) was precultured in MRS broth at 37°C for 24 h, and *S*. *Cerevisiae* (1.0×10^8^ CFU/mL) was precultured in malt extract medium in a rotary shaker (120 rpm) at 37°C for 24 h. The liquid inoculum was prepared by mixing the two inoculants at a ratio of 3:7, and they were then inoculated in sterilized dandelion. Fermentation time, temperature, inoculum concentration and moisture content are known to be the important factors for microbial growth and metabolite production under SSF conditions [19,20]. Therefore, these factors were selected for further optimization experiments with the target of increasing the dandelion flavonoid content.

#### 2.2.1 Experimental design

Single-factor experiments and response surface methodology were applied to optimize the fermentation conditions of dandelion. In single-factor experiments, fermentation time (36,48,72,96, and 120 h), fermentation temperature (30,33,35,37, and 40°C), inoculum concentration (5%,7.5%,10%, and 12.5% (v/w)) and moisture content (45%,50%,55%, and 60% (v/w)) were investigated for dandelion flavonoid content maximization. One factor was changed while the others were held constant in each experiment. The water in the inoculum was taken into account in the initial moisture content.

Box-Behnken design (BBD) was employed to determine the best combination of different variables to maximize the flavonoid content based on the single-factor experimental results. The proper range for fermentation time (A), fermentation temperature (B), inoculum concentration (C) and moisture (D) were preliminarily determined, and then response surface methodology was utilized to design the experimental project. The independent variables and their levels are given in Table 1. On the basis of the BBD data, a quadratic polynomial model was fitted to correlate the relationship between the independent variables and the response values to predict the optimized conditions. After fermentation, the dandelion was collected and allowed to dry (45°C, 24 h) for further extraction and determination of flavonoids.

**Table 1:** Experimental design and results for Box-Behnken.

Symbols	Independent variables	Levels		
		-1	0	1
A	Fermentation time (h)	36	48	72
B	Fermentation temperature (℃)	33	35	37
C	Inoculum concentration (%)	8	10	12
D	Moisture content (%)	50	55	60

#### 2.2.2 Determination of flavonoid content and preparation of crude extracts

Sample pretreatment: Fermented or unfermented dandelion was placed in a drying oven at 45°C for 24 h. A 1.0 g dandelion sample was mixed with 20.0 mL of distilled water in an 80°C water bath for 30 min, and the mixture was centrifuged at 5000 rpm for 15 min. The resulting supernatant was freeze-dried for determination of the flavonoid content.

Determination of flavonoid content: The flavonoid content was estimated by a colorimetric method with a few modifications [21,22]. Briefly, 200 mg of sample was redissolved in 20 mL of distilled water. Then, 2.5 mL of the flavonoid solution and 0.4 mL NaNO_2_ (5%, w/v) were mixed for 6 min, and then 0.4 mL of Al (NO_3_)_3_ (10%, w/v) was added and mixed. After 6 min, 2.0 mL of NaOH (1 M) was added. The solution was mixed thoroughly and incubated for 15 min. The absorbance was measured at 510 nm. The standard curve regression equation was y = 5.792x+0.044 (where y is absorbance and x is the rutin concentration in mg/mL), and R^2^ = 0.999.

Preparation of crude extracts: Water extracts (1.0 g, fermented or unfermented dandelion) were mixed with 35.0 mL of 40% ethanol at 70°C for 30 min, and the mixture was centrifuged at 5000 rpm for 15 min. The resulting supernatant was freeze-dried to obtain FDF and DF.

### 2.3 Compositional analysis of FDF and DF

#### 2.3.1 Sample preparation and extraction

A freeze-dried sample (DF or FDF) was crushed using a mixer mill (MM400, Retsch) with a zirconia bead for 1.5 min at 30 Hz. One hundred milligrams of powder was weighed and extracted overnight at 4°C with 1.0 mL of 70% aqueous methanol. Following centrifugation at 10,000 g for 10 min, the extracts underwent cleanup and preconcentration (CNWBOND Carbon-GCB SPE cartridge, 250 mg, 3 mL; ANPEL, Shanghai, China, www.anpel.com.cn/cnw) and filtered (SCAA-104, 0.22 μm pore size; ANPEL, Shanghai, China, http://www.anpel.com.cn/) before LC-MS analysis.

#### 2.3.2 HPLC conditions

The MRM was performed by Metware Biotechnology Co., Ltd. (Wuhan, China). The sample extracts were analyzed using a liquid chromatography electrospray ionization-tandem mass spectrometry (LC-ESI-MS/MS) system (HPLC, Shim-pack UFLC SHIMADZU CBM30A system, www.shimadzu.com.cn/; MS, Applied Biosystems 6500 Q TRAP, www.appliedbiosystems.com.cn/). The HPLC conditions were as follows: column, Waters ACQUITY UPLC HSS T3 C18 (1.8 μm, 2.1 mm*100 mm); solvent system, 0.04% acetic acid in water: 0.04% acetic acid in acetonitrile; gradient program, 100:0 V/V at 0 min, 5:95 V/V at 11.0 min, 5:95 V/V at 12.0 min, 95:5 V/V at 12.1 min, and 95:5 V/V at 15.0 min; flow rate, 0.40 mL/min; temperature, 40°C; and injection volume, 2μL. The effluent was alternatively connected to an ESI-triple quadrupole-linear ion trap (QTRAP)-MS.

#### 2.3.3 ESI-QTRAP-MS/MS

Linear ion trap (LIT) and triple quadrupole (QQQ) scans were acquired on the QTRAP equipped with an ESI Turbo Ion Spray interface, operating in positive ion mode and controlled by Analyst 1.6.3 software (AB Sciex). The ESI source operational parameters were as follows: ion source, turbo spray; source temperature, 500°C; ion spray (IS) voltage, 5500 V; ion source gas I (GSI) and gas II (GSII), and curtain gas (CUR), 55, 60, and 25.0 psi, respectively; and collision gas (CAD), high. Instrument tuning and mass calibration were performed with 10 and 100 μmol/L polypropylene glycol solutions in QQQ and LIT modes, respectively. QQQ scans were acquired as multiple reaction monitoring (MRM) mode experiments with the collision gas (nitrogen) set to 5 psi. The declustering potential (DP) and collision energy (CE) for individual MRM transitions were optimized [23]. A specific set of MRM transitions was monitored for each period according to the metabolites eluted within that period.

### 2.4 *In vitro* antioxidant activity of FDF and DF

#### 2.4.1 DPPH radical-scavenging activity

The DPPH assay was conducted following a reported method with some modification [24,25]. Briefly, a 2×10^−4^ mol/L DPPH solution was prepared using 95% ethanol, and 1 mL of the DPPH solution was added to 1 mL of sample solutions at different concentrations (FDF or DF). BHA was used as the positive control. The absorbance of the mixtures was measured at a wavelength of 517 nm using a spectrophotometer. The DPPH radical-scavenging activity was calculated using the following equation:

$$\text{DPPH}\;\text{radical}-\text{scavenging}\;\text{activity}\;(\%)=(1-\frac{{\text{A}}_{s}-{\text{A}}_{\text{b}}}{{\text{A}}_{d}})\times \text{100}\%,$$ (1)

where A_d_ is the absorbance of the mixture containing DPPH and ethanol, A_s_ is the absorbance of the mixture containing the sample and DPPH, and A_b_ is the absorbance of the mixture containing the sample and ethanol.

#### 2.4.2 Measurement of reducing power

The reducing power assay was conducted according to a previously published method [26] with slight modification. Briefly, 0.75 mL of samples (FDF or DF) at different concentrations were mixed with 0.75 mL of phosphate buffer (0.2 M, pH 6.6) and 0.75 mL of 1% potassium ferricyanide. The mixture was warmed for 20 min at 50°C, and then 0.75 mL of 10% TCA was added; the mixture was then centrifuged at 3000 g for 10 min. Next, 1.5 mL of the supernatant was mixed with 1.5 mL water and 400 μL of 0.1% FeCl_3_. The absorbance of the mixture was measured at 700 nm, and BHA was used as a positive control.

### 2.5 Statistical analysis

Response surface test design and analysis were conducted with Design-Expert 8.0.6 software (Stat-Ease Inc., US). All experiments were performed in triplicate. The data are expressed as the mean ± standard deviation (S.D.), and one-way ANOVA followed by Duncan’s multiple range tests were used to evaluate significant differences. The differences were accepted as significant at *P*<0.05.

Qualitative and quantitative analyses of metabolites followed the methods of Wang and Fraga. Based on the self-built database MWDB (Metware Biotechnology Co., Ltd. Wuhan, China) and the public database of metabolite information, the qualitative analysis of the primary and secondary spectral data of mass spectrometry was performed.

## 3. Results and discussion

### 3.1. Optimization of dandelion fermentation conditions by response surface methodology

#### 3.1.1. Single-factor experimental results

**Fig 1:**
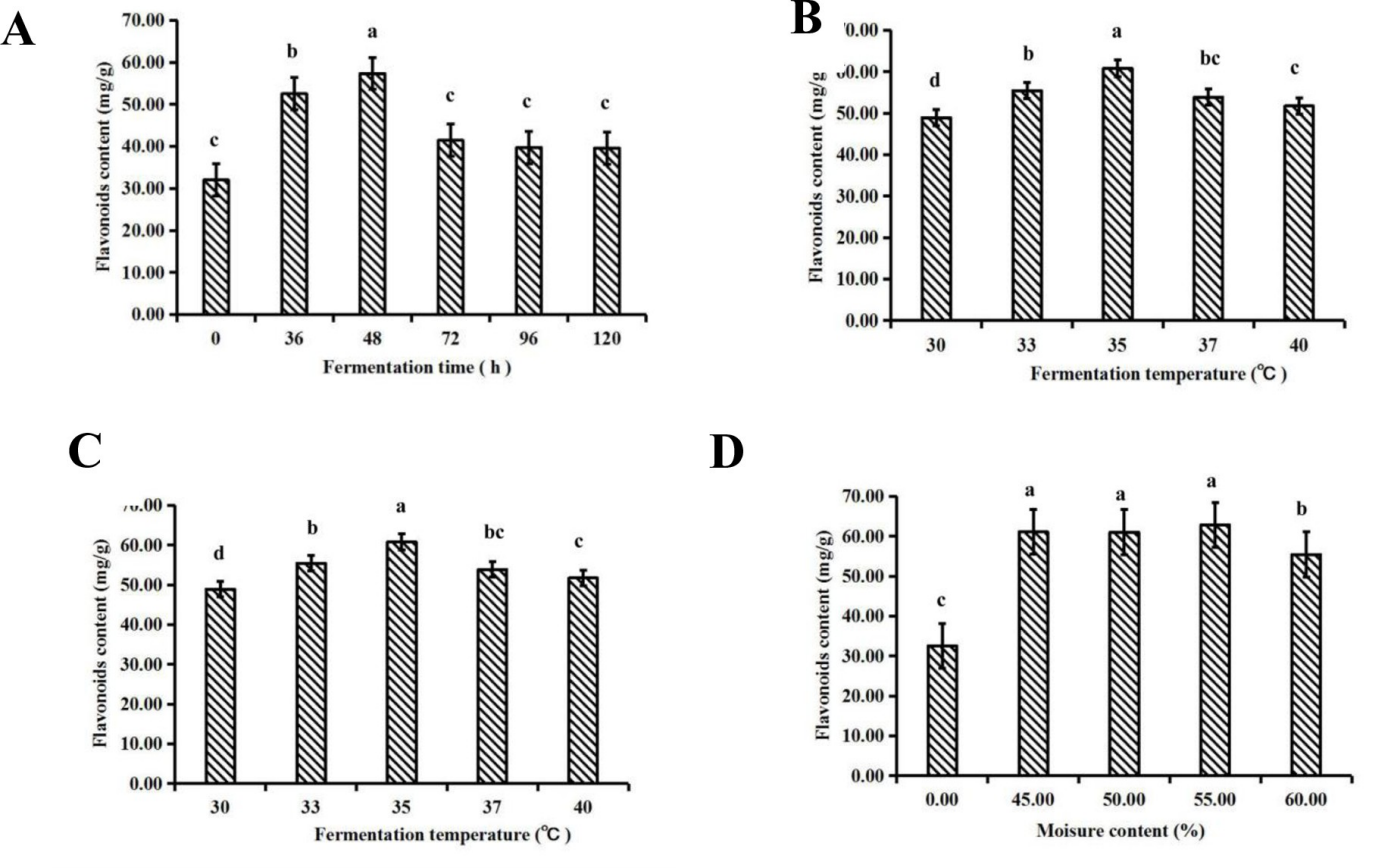
Effect of different fermentation conditions on the flavonoid content of dandelion and columns not sharing a common letter showed significant difference (*P*<0.05). (A) Fermentation time, h. (B) Fermentation temperature, ℃. (C) Inoculum concentration, %. (D) Moisture content, %.

The effect of fermentation time on flavonoid content is presented in Fig 1A. The flavonoid content increased with increasing fermentation time, and the maximum concentration was recorded at 48 h. Then, at longer fermentation times, the flavonoid content decreased. Thus, 48 h was taken as the central fermentation time point for the response surface optimization test. With increasing fermentation temperature, the flavonoid content first increased and then decreased (Fig 1B). The optimum fermentation temperature was found to be 35°C, which yielded a content of 60.80 mg/g. Thus, 35°C was utilized as the central fermentation temperature point for the response surface optimization test. Fig 1C shows that the flavonoid content increased with increasing inoculum concentration, and there was no remarkable difference between the groups (*P*>0.05). An inoculum concentration of 10% was taken as the center point for the response surface optimization test. The highest flavonoid content (62.84 mg/g) was observed at a 55% moisture content (Fig 1D), so this moisture content was taken as the central point for the response surface optimization test.

#### 3.1.2. Response surface analysis

Table 2 shows the fermentation conditions and resulting flavonoid contents according to the factorial design. By applying multiple regression analysis on the experimental data, the flavonoid content and the independent variables were related by the following fitted second-order polynomial equation:

$$\text{Y}=-4951.68479+5.83405\text{A}+194.84759\text{B}+31.75407\text{C}+46.60488\text{D}-0.058477\text{AB}-0.091545\text{AC}+0.012849\text{AD}+0.76220\text{BC}-0.040385\text{BD}-0.77081\text{CD}-0.032156{\text{A}}^{2}—2.81303{\text{B}}^{2}-0.52317{\text{C}}^{2}-0.34733{\text{D}}^{2},$$ (2)

**Table 2:** Experimental design and results for Box-Behnken.

No.	A(fermentation time)	B(fermentation temperature)	C(inoculum concentration)	D(moisture content)	Y(flavonoids content, mg/g)
1	-1	-1	0	0	42.78
2	1	0	0	-1	45.54
3	0	-1	-1	0	49.97
4	0	0	0	0	60.17
5	0	0	0	0	64.81
6	-1	0	0	-1	46.99
7	-1	1	0	0	44.66
8	1	1	0	0	42.60
9	0	0	-1	1	64.39
10	0	1	0	-1	43.91
11	0	-1	0	-1	42.53
12	1	0	1	0	54.61
13	0	0	0	0	64.89
14	0	0	0	0	65.30
15	-1	0	-1	0	41.07
16	0	0	1	1	49.77
17	-1	0	1	0	52.79
18	-1	0	0	1	40.00
19	0	0	-1	-1	46.19
20	0	1	0	1	44.09
21	0	0	1	-1	62.40
22	0	0	0	0	66.72
23	0	1	-1	0	47.76
24	0	-1	1	0	45.51
25	1	0	0	1	43.18
26	0	1	1	0	55.50
27	1	-1	0	0	49.13
28	1	0	-1	0	56.08
29	0	-1	0	1	44.33

The analysis of variance for the experimental results of the BBD is summarized in Table 3. The P-value of the model (*P*<0.0001) was significant, and the lack of fit (*P* = 0.2077) was non-significant, which indicated that the model was a good fit with the experimental data. Moreover, the R^2^ of the model was 0.9178, which indicated that the experimental data of the response surface methods had a high degree of accuracy and reliability and that the predicted data correlated well. Furthermore, the content of flavonoids was significantly affected by fermentation time (*P*< 0.05).

**Table 3:** ANOVA for the fitted regression model.

Source	Sum of Squares	df	Mean Squares	F value	P value
Model	1909.34	14	136.38	11.17	<0.0001
A	43.51	1	43.51	3.56	0.0400
B	1.53	1	1.53	0.13	0.7286
C	19.04	1	19.04	1.56	0.2323
D	0.26	1	0.26	0.022	0.8850
AB	17.73	1	17.73	1.45	0.2482
AC	43.44	1	43.44	3.56	0.0802
AD	5.35	1	5.35	0.44	0.5188
BC	37.18	1	37.18	3.05	0.1029
BD	0.65	1	0.65	0.053	0.8205
CD	237.66	1	237.66	19.47	0.0006
A^2^	704.07	1	704.07	57.67	<0.0001
B^2^	821.26	1	821.26	67.27	<0.0001
C^2^	28.41	1	28.41	2.33	0.1494
D^2^	489.09	1	489.09	40.06	<0.0001
Residual	170.92	14	12.21		
Lack of fit	146.44	10	14.64	2.39	0.2077
Pure error	24.48	4	6.12		
Cor total	2080.26	28			

**Fig 2:**
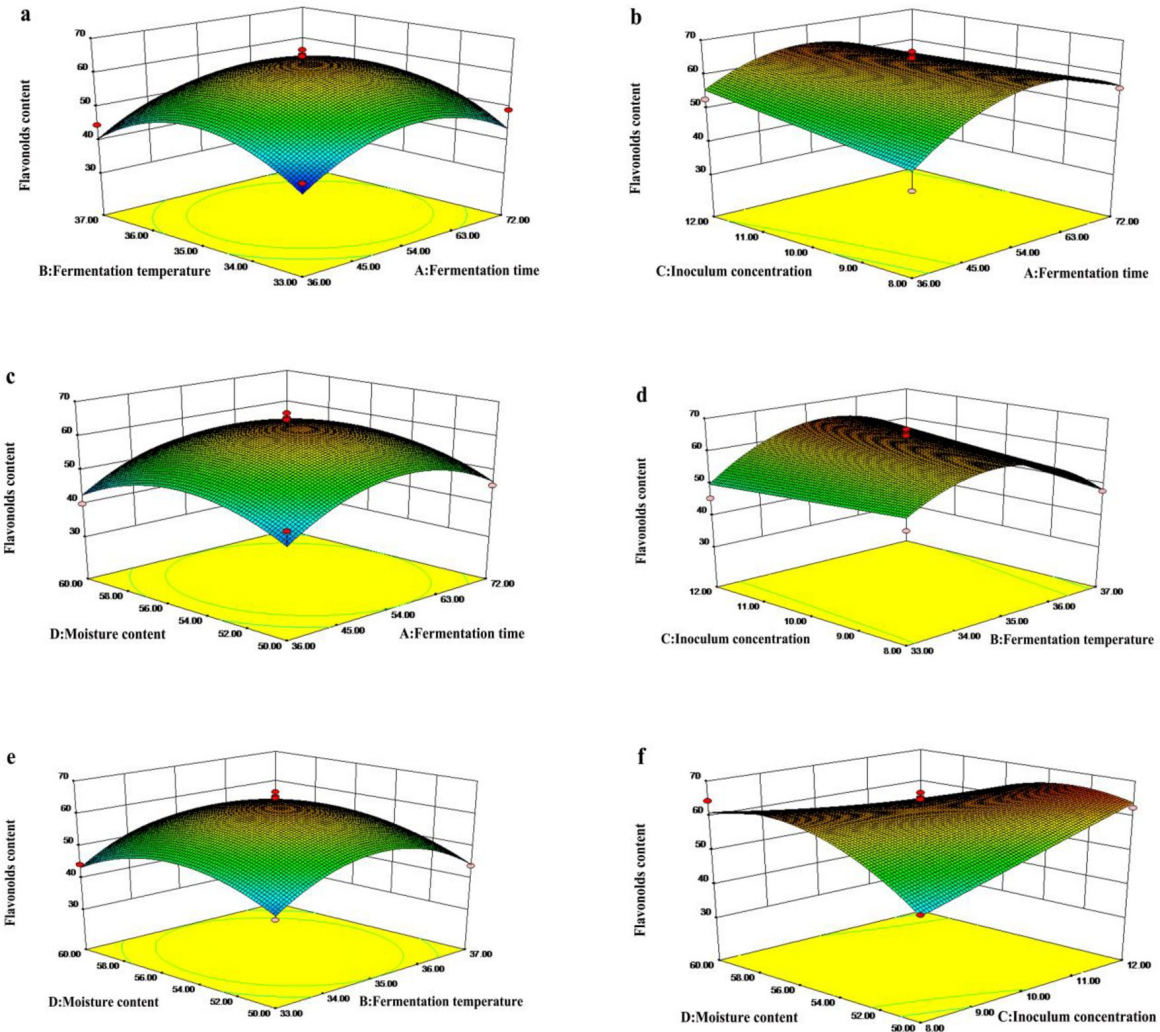
Response surface (3D) for the effect of different fermentation conditions (A: fermentation time, h; B: extraction temperature, ℃; C: inoculum concentration, % and D: moisture content, %) on the response Y (flavonoids content, mg/g).

Response surface plots of flavonoid content are shown in Fig 2A–2F. The interaction effect of fermentation time and fermentation temperature on the content of flavonoids is shown in Fig 2A. With increasing fermentation time, the flavonoid content of fermented dandelion first increased and then decreased. There was a similar trend in fermentation temperature. The results showed that the flavonoid content was not significantly affected by the interaction (*P*>0.05). Moreover, interactions between fermentation time and inoculum concentration, fermentation time and moisture content, fermentation temperature and inoculum concentration, and fermentation temperature and moisture content also had no significant effect on the flavonoid content of fermented dandelion (Fig 2B–2E). Fig 2F shows the interaction effect of inoculum concentration and moisture content on the content of flavonoids. The flavonoid content increased with increasing inoculum concentration. When the inoculum concentration was fixed, the flavonoid content first increased with increasing moisture content and then decreased. The interaction between inoculum concentration and moisture content showed a significantly positive effect on the flavonoid content of fermented dandelion (*P*< 0.05).

It could be concluded that the optimal fermentation conditions to maximize the flavonoid content of fermented dandelion were a fermentation time of 52.04 h, a fermentation temperature of 35.34°C, an inoculum concentration of 12.00%, and a moisture content of 52.69% by the regression equation (Eq. (2)) obtained using Design-Expert 8.0.6 software. The predicted flavonoid content under the above conditions was 65.73 mg/g. A verification experiment was performed with the optimum fermentation conditions (fermentation time: 52 h; fermentation temperature: 35°C; inoculum concentration: 12%; and moisture content: 52%), and the flavonoid content of fermented dandelion was 66.05 ± 1.89 mg/g (N = 5). The verification value differed by 0.49% from the predicted value, indicating that the model was reliable and accurate.

**Fig 3:**
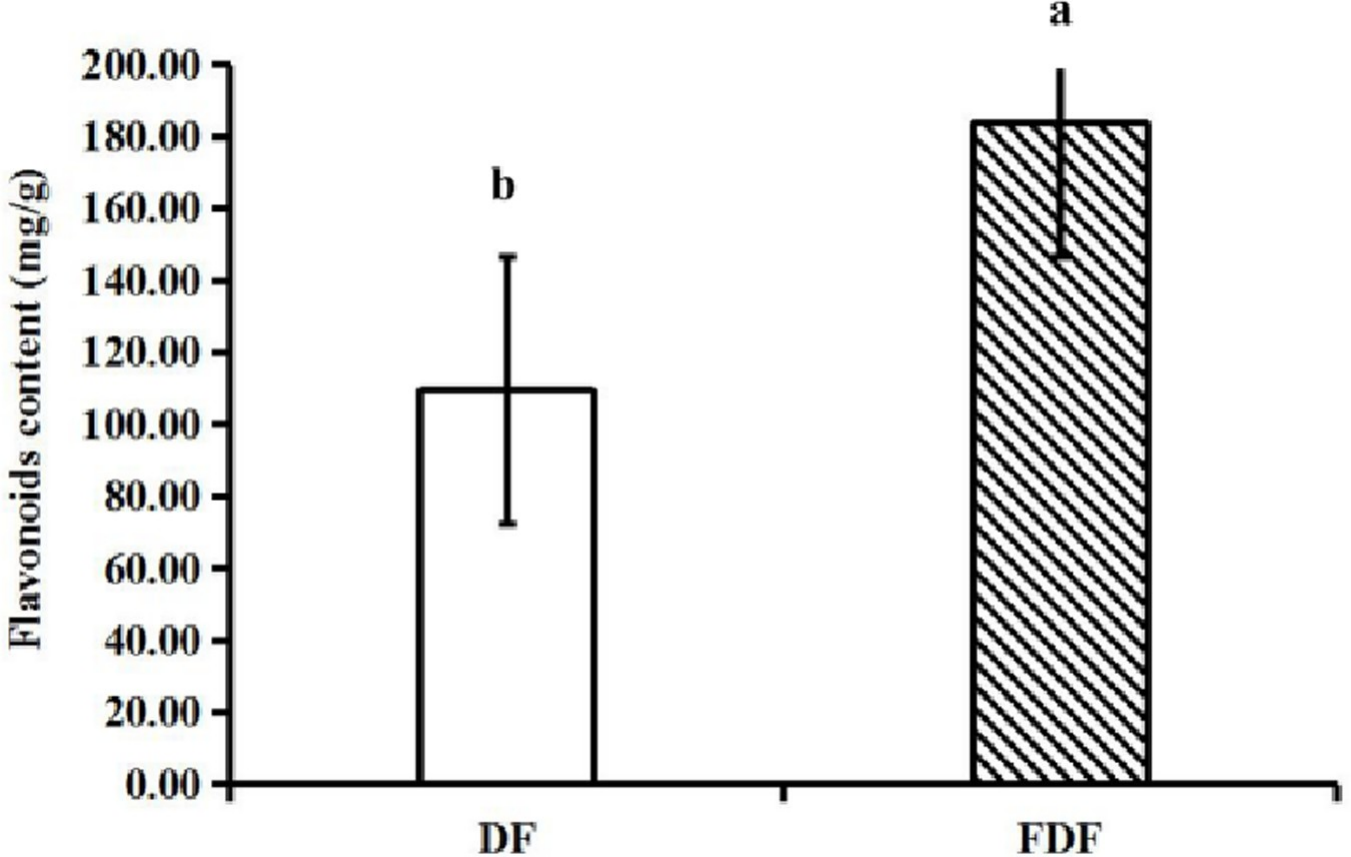
The flavonoids content of crude extracts before and after fermentation and columns not sharing a common letter showed significant difference (*P*<0.05).

We can see the effect of fermentation on the flavonoid content of crude extracts in Fig 3. The flavonoid content of FDF (183.72±2.24 mg/g) was significantly higher than that of DF (109.49±1.05 mg/g) (*P*<0.05). The flavonoid content of FDF increased by 67.80% compared with that of DF. The results clearly indicate that the fermentation process significantly improved the flavonoid content of dandelion. This conclusion is in agreement with that of a previous report by Kim et al [27]. Screened *Lactobacillus acidophilus*F-46 was inoculated in sterilized dandelion extract to prepare a fermented dandelion beverage. They found that the total flavonoid content of the fermented dandelion beverage samples was significantly higher than that of nonfermented dandelion beverage samples. Our results clearly indicate that the SSF process applied in this study increased the flavonoid content of dandelion relative to that of unfermented samples.

### 3.2. Differential composition in response to DF and FDF

**Fig 4:**
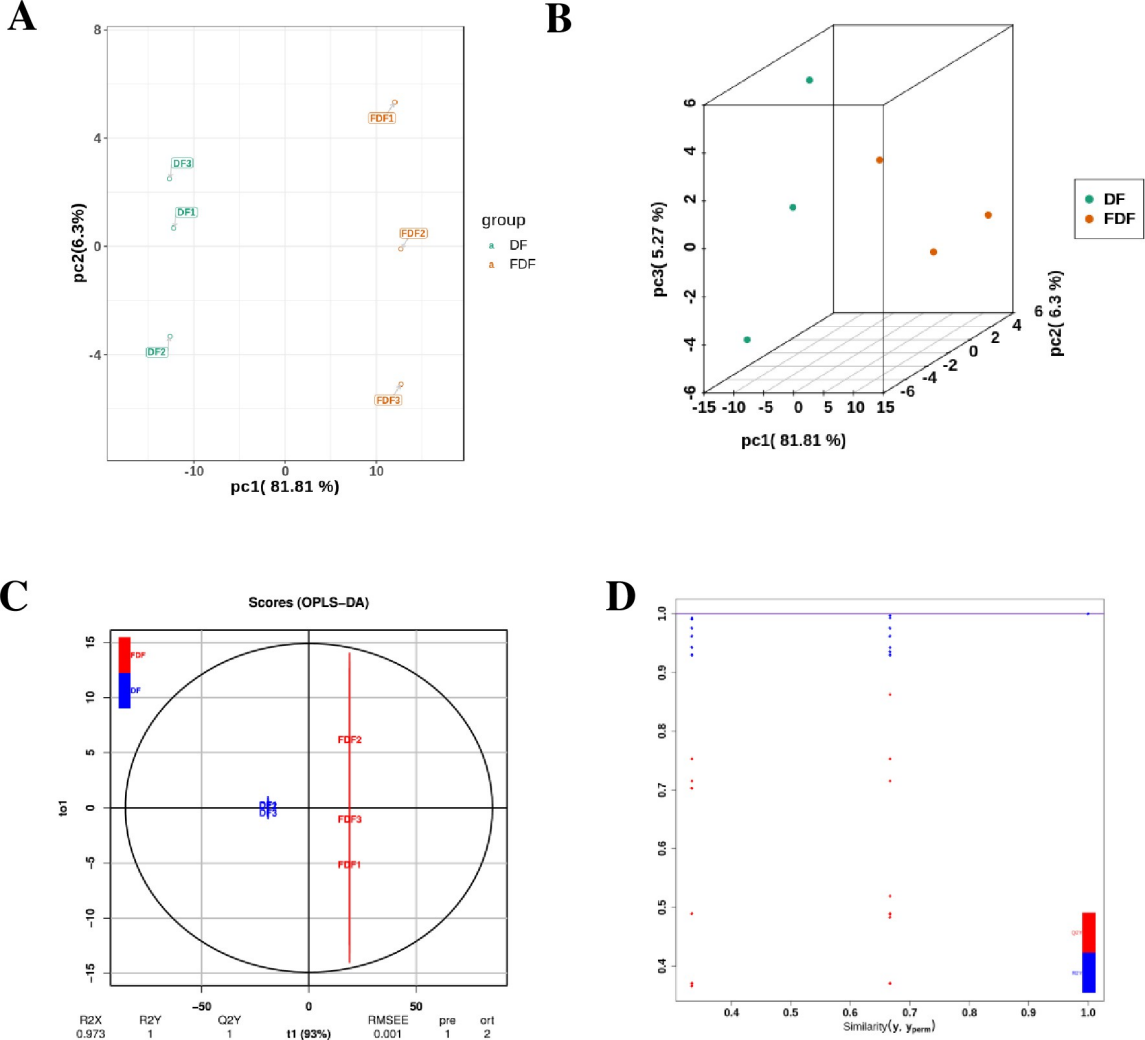
Flavonoids metabolic profile exposed to fermented dandelion and unfermented dandelion obtained through UPLC-ESI-MS/MS-based metabolomics and multivariate data analysis. (A) The two-dimensional scatter plot of the PCA model of two groups. (B)The three-dimensional scatter plot of the PCA model of two groups. (C) The scatter plot of the OPLS-DA model. (D) 200-permutation test was further applied to validate the OPLS-DA model, confirming the robustness of the model.

To explore flavonoid metabolites before and after fermentation, metabolomic analysis was carried out. Identified compounds m/z of parent ions are shown in S1 Fig. To improve the reliability of the results of this experiment, quality control samples were tested. Typical total ion current chromatograms (TICs) of all samples are shown in S2 Fig. The uniformity of the retention time in each group indicated the stability of the instrument and the reliability of the data. After the data were acquired, PCA was used to determine independence characteristics and remove noise from the data. The two-dimensional and three-dimensional scatter plots of the PCA model of the two groups are shown in Fig 4A and 4B. As shown in the chart, there was better repeatability within each group than between groups, and there was a significant difference between FDF and DF, suggesting that the content of flavonoids can be altered by fermentation.

The different metabolites between the FDF and DF groups were the key to explaining why good fermentation technology is important for substance optimization. Furthermore, supervised OPLS-DA was applied and is shown in Fig 4C. This model filters orthogonal signals with regard to the Y variable (the response variable) and leaves the signals that are highly correlated to the Y variable [28] to ensure a high fitting degree of the models. The parameters of the value of the first predicted component (R^2^Y = 1) and the value for the predictability of the model (Q^2^ = 1) indicated that the OPLS-DA model can distinguish FDF and DF well. A test of 200 permutations was further applied to validate the model shown in Fig 4D. The R^2^ and Q^2^ intercept values in the FDF vs DF model were lower than those in the original model, and the low Q^2^ intercept value indicates the robustness of the model. Thus, we could use variable importance in project (VIP) to select differential metabolites, and a VIP value exceeding 1.0 was set as a selection criterion.

**Fig 5:**
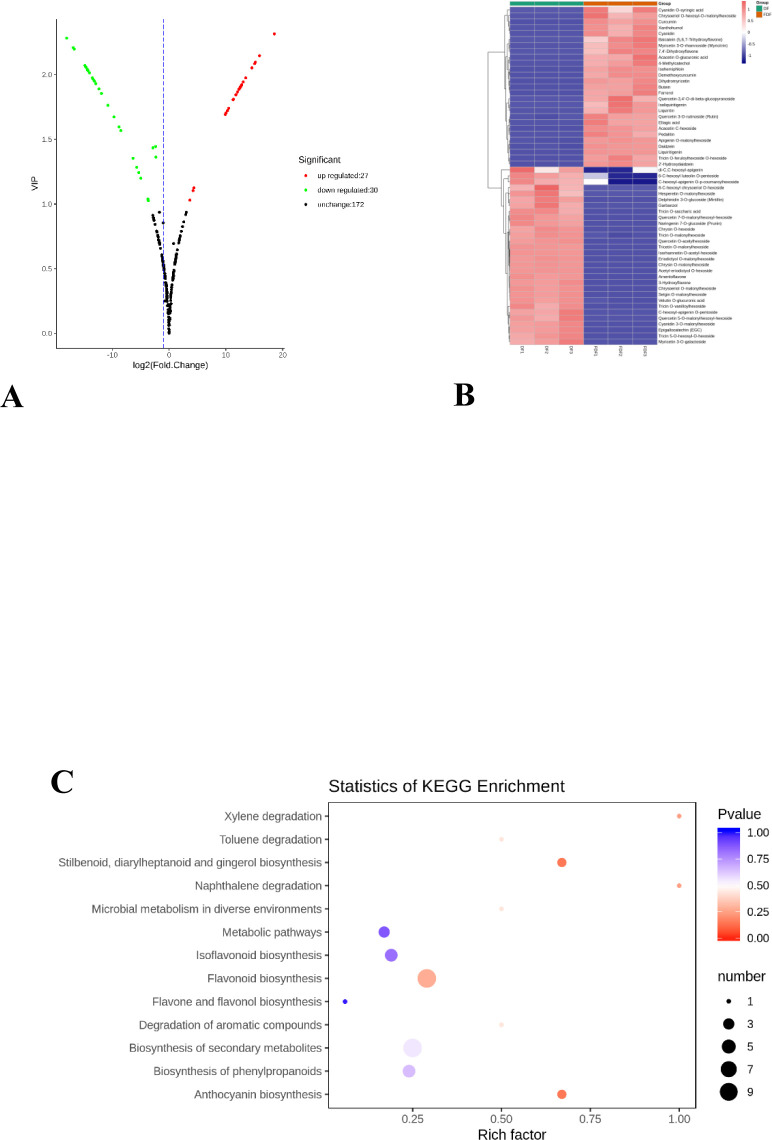
Differential metabolite screening and functional annotation. (A) Differentially expressed metabolites‘ volcano plot for group FDF vs DF. (B) Differentially expressed metabolites’ heatmap of hierarchical clustering analysis for group FDF vs DF. (C) The pathway enrichment of differential metabolite.

Differential flavonoid metabolites were screened for each comparison group by combining the fold change and VIP values of the OPLS-DA model. The criteria for screening included a fold change value ≥ 2 or ≤ 0.5 and a VIP value ≥ 1. To quickly examine the differences in metabolite expression levels between the two groups and the statistical significance of the differences, the differential metabolites were shown in a volcano plot. In Fig 5A, black dots represent the metabolites detected but not significantly different, and the green dots and red dots represent significantly downregulated metabolites and upregulated metabolites, respectively. In detail, there were 57 significantly different flavonoid metabolites (27 upregulated, 30 downregulated), and 172 were unchanged. All differential metabolite profiles in response to fermentation are displayed in a heat map (Fig 5B). In the heat map, the content of flavonoid metabolites in FDF compared with those in DF varied greatly. The differential flavonoid metabolites from the two groups were annotated by the Kyoto Encyclopedia of Genes and Genomes (KEGG) database. Furthermore, 25 annotated metabolites of all 57 detected differential metabolites were mapped to their respective biochemical pathways as described in the KEGG database, and enrichment factors of these pathways were calculated by MetaboAnalyst 3.0. The pathway enrichment results are shown in Fig 5C. The analysis revealed that the metabolites that changed in response to fermentation participated in 13 target pathways, and the size of the points in the figure represents the number of significantly different metabolites enriched on the corresponding pathways. The first four highest impact pathways were anthocyanin biosynthesis stilbenoid, diarylheptanoid and gingerol biosynthesis; naphthalene degradation; and xylene degradation.

**Table 4:** Relative content of 25 annotated differential metabolites in DF and FDF.

Compounds	Class	Relative content		VIP	LogFC
		DF	FDF		
Quercetin 3-O-rutinoside (Rutin)	Flavonol	172333	2196667	1.0302	3.6720
Amentoflavone	Flavone	197000	-	2.0421	-14.4179
Daidzein	Isoflavone	31267	648667	1.1254	4.3748
Naringenin 7-O-glucoside (Prunin)	Flavanone	86133	-	1.9557	-13.2244
4-Methylcatechol	Polyphenol	-	23200	1.8098	11.3319
Delphinidin 3-O-glucoside (Myrtillin)	Anthocyanins	230000	-	2.0576	-14.6413
Baicalein (5,6,7-Trihydroxyflavone)	Flavone	-	59633	1.9136	12.6939
Epigallocatechin (EGC)	Polyphenol	80733	-	1.9487	-13.1310
Myricetin 3-O-rhamnoside (Myricitrin)	Flavonol	-	32100	1.8462	11.8004
Ellagic acid	Polyphenol	-	52333	1.9014	12.5055
Curcumin	Polyphenol	-	103800	1.9755	13.4935
Dihydromyricetin	Flavonol	-	3430000	2.3157	18.5399
3-Hydroxyflavone	Flavonol	15467	-	1.7631	-10.7469
Isoliquiritigenin	Flavanone	-	8757	1.6929	9.9262
Liquiritigenin	Flavanone	-	55367	1.9081	12.5868
2'-Hydroxydaidzein	Isoflavone	-	76900	1.9435	13.0608
Butein	Flavanone	-	22267	1.8054	11.2727
Xanthohumol	Flavanone	-	9540	1.7043	10.0498
7,4'-Dihydroxyflavone	Flavone	-	52133	1.9001	12.5000
Cyanidin	Anthocyanins	-	566000	2.1469	15.9405
Garbanzol	Flavanone	34567	-	1.8550	-11.9072
Pedalitin	Flavonoid	-	63700	1.9228	12.7891
Demethoxycurcumin	Polyphenol	-	48300	1.8928	12.3898
Farrerol	Flavonoid	-	46200	1.8878	12.3257
Liquiritin	Flavonoid	-	340000	2.0965	15.2053

The annotated differential metabolites are listed in Table 4. The results of differential metabolites were analyzed, and we found that SSF can effectively enhance dandelion flavonoids, such as cyanidin, curcumin, dimethoxy curcumin, quercetin 3-O-rutinoside (rutin) and dihydromyricetin. Cyanidin and delphinidin 3-O-glucoside (myrtillin) are anthocyanins and are involved in the anthocyanin biosynthesis pathway. Anthocyanins, which are natural compounds known to be powerful antioxidants, have attracted attention [29]. The relative content of cyanidin in FDF significantly increased compared to that in DF, but that of delphinidin 3-O-glucoside significantly decreased, so fermentation changed the color of the dandelion material. Curcumin and demethoxycurcumin are polyphenolic compounds involved in the stilbenoid, diarylheptanoid and gingerol biosynthesis pathway. Demethoxycurcumin is a naturally occurring derivative of curcumin. Previous work has shown that curcumin has various pharmacological activities, such as antioxidant, antiviral, antibacterial, anti-inflammatory, and anticancer properties [30,31]. Rutin and dihydromyricetin are flavonols, and their relative contents were highest among the annotated differential metabolites. Rutin was detected in DF and FDF, but dihydromyricetin was detected only in FDF. Rutin is a glycoside of quercetin and has shown antioxidant properties and several pharmacological functions, such as vasoprotective, antiproliferative, antithrombotic and cardioprotective activities [32]. Dihydromyricetin is a very active flavonoid and exhibits antioxidant activity and hepatoprotective effects [33]. The above results indicate that fermentation not only improved the content of pre-existing flavonoid metabolites in dandelion but also promoted the synthesis of new flavonoid metabolites. Further research is needed to explore how the fermentation process influences the abundance of flavonoid metabolites. As we observed, these differential metabolites are closely related to each other in structural and biochemical terms, which could explain why they increased during the fermentation process.

### 3.3. *In vitro* antioxidant activity

**Fig 6:**
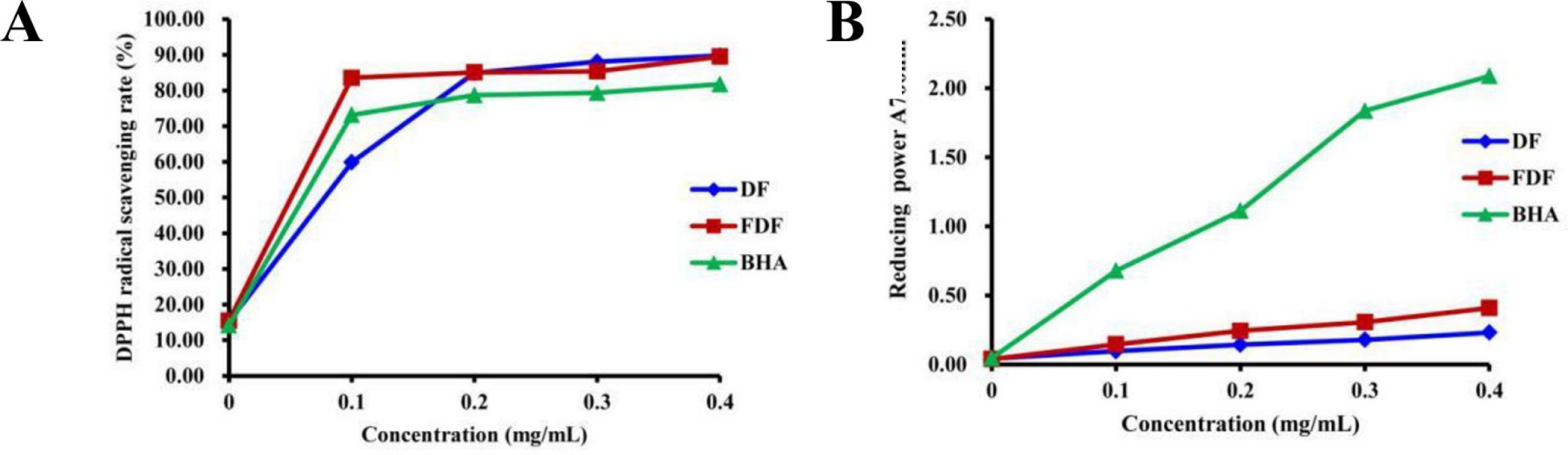
Antioxidant activities of FDF and DF *in vitro*. (A) DPPH radical-scavenging capacity. (B) Reducing power assay.

DPPH is stable and easy to handle and has been accepted for evaluating the radical-scavenging activity of antioxidants from plant extracts [34,35]. As shown in Fig 6A, the DPPH radical-scavenging capacity of FDF and DF were enhanced with increasing concentrations. FDF exhibited significantly higher DPPH radical-scavenging activity than those of DF and BHA at a concentration of 0.1 mg/mL (*P*<0.05). The IC50 values of DPPH radical-scavenging activity were found to be 0.075 and 0.088 mg/mL for FDF and DF, respectively, indicating the greater antioxidant activity of FDF than that of DF. Fig 6B shows that the reducing power of FDF was correlated with the concentration. FDF had stronger reducing power than that of DF, but the efficiency was lower than that of BHA at all the concentrations tested in this study. When the concentration was 0.4 mg/mL, the reducing power of FDF, DF and BHA was 0.41±0.01, 0.23±0.01 and 2.10±0.13, respectively. The results revealed that the reducing power of FDF was significantly higher than that of DF, which might have been caused by fermentation. As mentioned above, FDF had a stronger antioxidant activity, which could be explained by the generation of flavonoids through fermentation that improved the content of flavonoids in dandelion.

## 4. Conclusions

In conclusion, the solid-state fermentation conditions of dandelion were optimized by response surface methodology to increase the flavonoid content of dandelion and crude extracts. Differential flavonoid metabolites before and after fermentation were detected by UPLC-ESI-MS/MS. Changes in flavonoid metabolites between unfermented dandelion crude extracts and fermented dandelion crude extracts changed notably, and the differential metabolite results illustrated that solid-state fermentation can effectively enhance the content of dandelion flavonoids. In addition, fermented dandelion crude extracts displayed superior antioxidant activity to that of unfermented dandelion crude extracts *in vitro*. Hence, solid-state fermentation improved the bioavailability of flavonoids from dandelion, which can be used to prepare natural antioxidants, functional foods or food additives. However, the in vivo functional properties of fermented dandelion crude extracts are still unclear, and research into its in vivo structure-activity relationship is ongoing.

## Supporting information

S1 Fig The mass charge ratio (m/z) of significant difference metabolites. (DOCX)

S2 Fig Typical total ion current chromatograms (TIC) of all samples. (DOCX)
