# Supplementary figures and images for: The effects of solid-state fermentation on the content, composition and in vitro antioxidant activity of flavonoids from dandelion
Source: PLoS One. 2020 Sep 15;15(9):e0239076. doi: 10.1371/journal.pone.0239076 (PMC7491732; doi:10.1371/journal.pone.0239076)

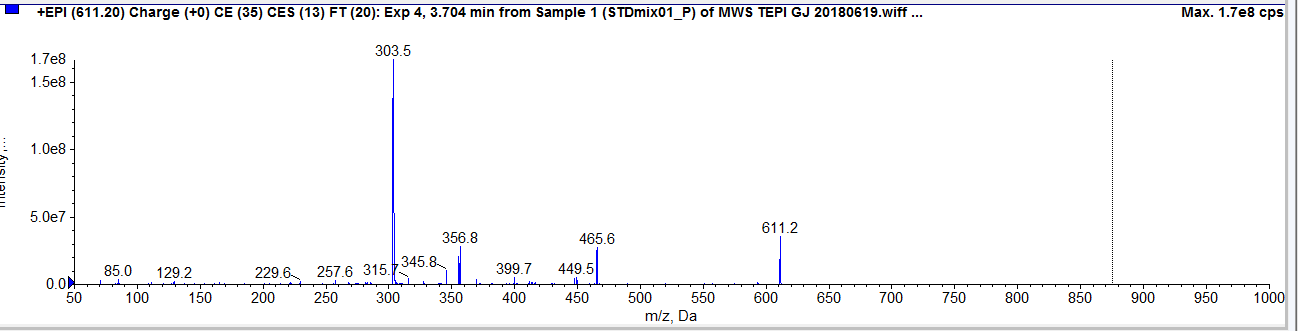


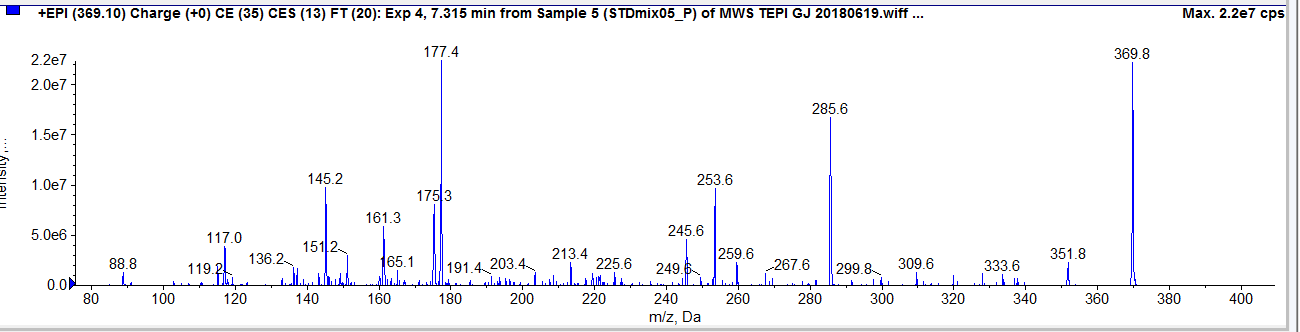


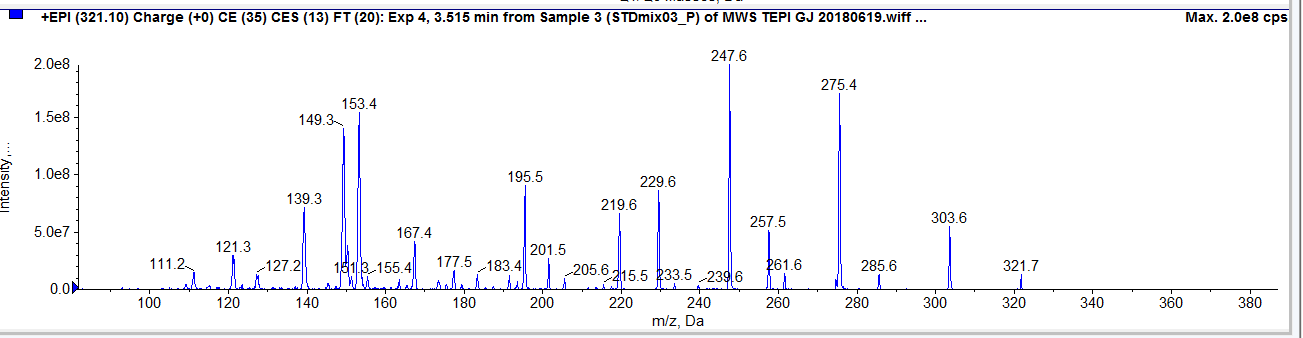


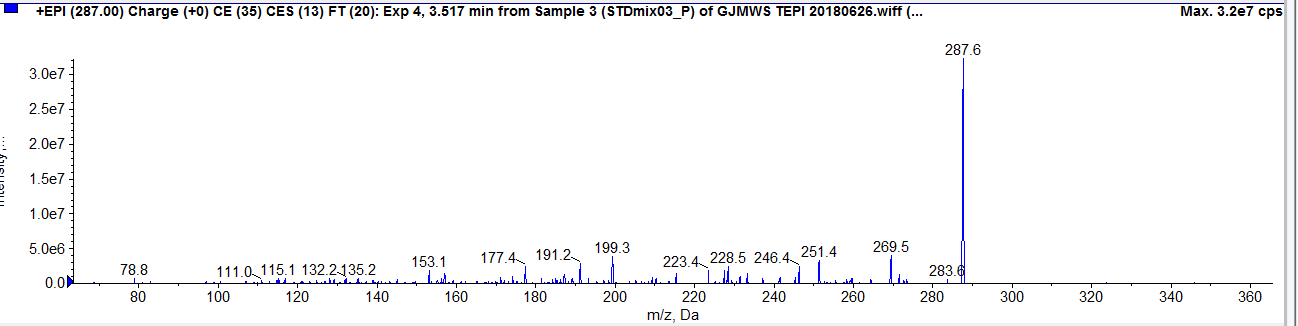


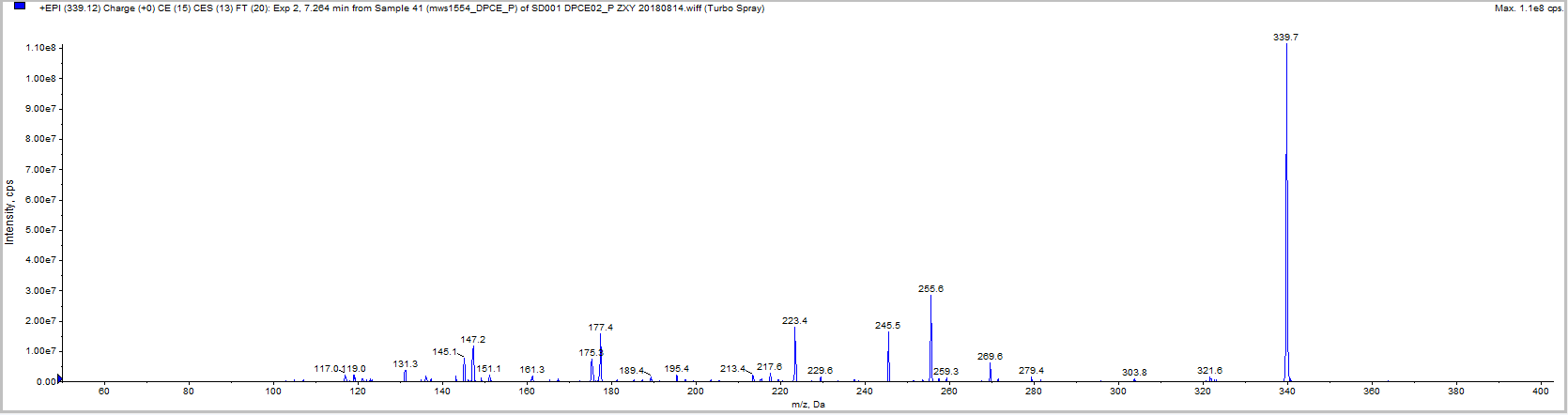


**S1-S5 Figs. The mass charge ratio (m/z) of significant difference metabolites.**

Supplement: S1 Fig — (DOCX) [file pone.0239076.s001.docx]

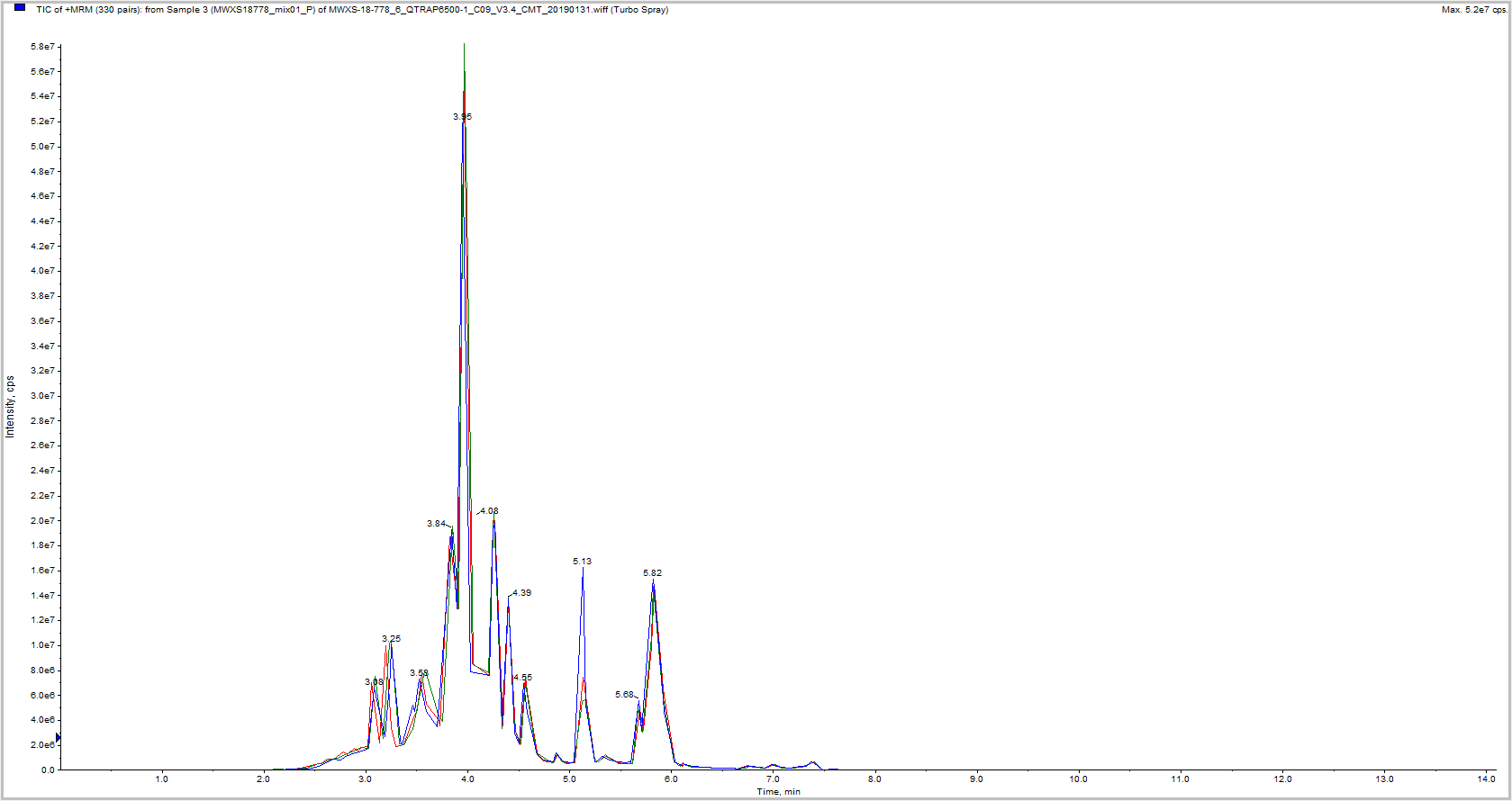


**S6 Fig: Typical total ion current chromatograms (TIC) of all samples.**

Supplement: S2 Fig — (DOCX) [file pone.0239076.s002.docx]
